# 1-(4-Bromo­benz­yl)-2-(4-bromo­phen­yl)-1*H*-benzimidazole

**DOI:** 10.1107/S1600536814009076

**Published:** 2014-04-26

**Authors:** Hua-Jun Ma, Zhi-Rong Qu

**Affiliations:** a Key Laboratory of Organosilicon Chemistry and Material Technology of the Ministry of Education, Hangzhou Normal University, No. 58, Haishu Road, Hangzhou, 311121, People’s Republic of China

## Abstract

There are two mol­ecules in the asymmetric unit of the title compound, C_20_H_14_Br_2_N_2_. In the first, the dihedral angles between the mean plane of the benzimidazole group and those of the 4-bromo­benzyl and 4-chloro­phenyl groups are 50.72 (17) and 71.29 (16)°, respectively, while the corresponding angles in the second mol­ecule are 42.09 (16) and 89.05 (17)°. The 4-bromo­benzyl and 4-bromo­phenyl groups make an angle of 68.1 (2) and 85.1 (21)° with each other in the two mol­ecules. In the crystal, weak C—H⋯N and C—H⋯Br hydrogen bonds link the mol­ecules along the *c*-axis direction. Br⋯Br inter­actions [3.5733 (9)Å] are also observed.

## Related literature   

For the chemistry of benzimidazoles, see: Steel (1990 ▶); Bhattacharya & Chaudhuri (2008 ▶); Horton *et al.* (2003 ▶); Boiani & González (2005 ▶); Bai *et al.* (2001 ▶); Hasegawa *et al.* (1999 ▶); Bouwman *et al.* (1990 ▶); Pujar & Bharamgoudar (1988 ▶). For their use in sunthesis, see: Sasaki *et al.* (1991 ▶); Wan *et al.* (2009 ▶).
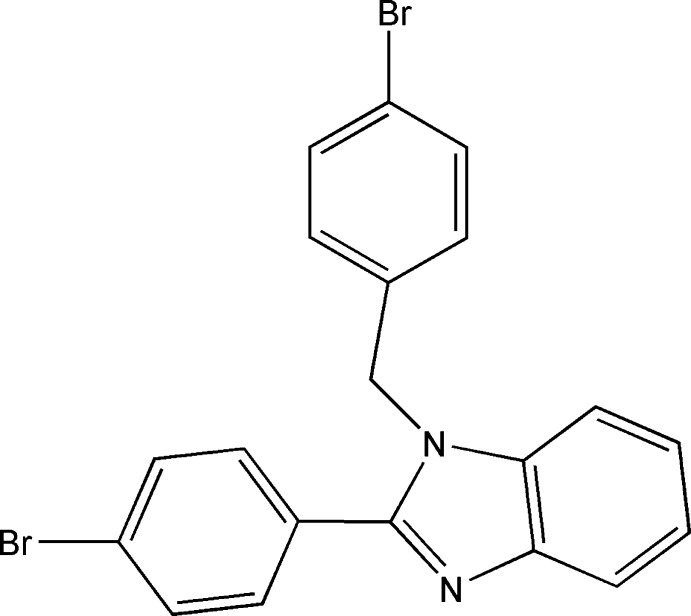


## Experimental   

### 

#### Crystal data  

C_20_H_14_Br_2_N_2_
*M* *_r_* = 442.15
Triclinic, 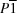

*a* = 9.7537 (9) Å
*b* = 10.5758 (10) Å
*c* = 17.8255 (17) Å
α = 83.435 (2)°
β = 81.702 (2)°
γ = 75.621 (2)°
*V* = 1756.6 (3) Å^3^
*Z* = 4
Mo *K*α radiation
μ = 4.62 mm^−1^
*T* = 293 K
0.30 × 0.26 × 0.20 mm

#### Data collection  

Bruker APEXII CCD diffractometer
Absorption correction: multi-scan (*SADABS*; Bruker, 2004 ▶) *T*_min_ = 0.258, *T*_max_ = 0.398
22195 measured reflections
8094 independent reflections
5171 reflections with *I* > 2σ(*I*)
*R*_int_ = 0.034

#### Refinement  

*R*[*F* ^2^ > 2σ(*F* ^2^)] = 0.041
*wR*(*F* ^2^) = 0.128
*S* = 1.03
8094 reflections
433 parameters
H-atom parameters constrained
Δρ_max_ = 0.78 e Å^−3^
Δρ_min_ = −0.51 e Å^−3^

### 

Data collection: *APEX2* (Bruker, 2004 ▶); cell refinement: *SAINT* (Bruker, 2004 ▶); data reduction: *SAINT*; program(s) used to solve structure: *SHELXS97* (Sheldrick, 2008 ▶); program(s) used to refine structure: *SHELXL97* (Sheldrick, 2008 ▶); molecular graphics: *SHELXTL* (Sheldrick, 2008 ▶); software used to prepare material for publication: *SHELXTL*.

## Supplementary Material

Crystal structure: contains datablock(s) I, New_Global_Publ_Block. DOI: 10.1107/S1600536814009076/bx2457sup1.cif

Structure factors: contains datablock(s) I. DOI: 10.1107/S1600536814009076/bx2457Isup2.hkl

Supporting information file. DOI: 10.1107/S1600536814009076/bx2457Isup3.cml

CCDC reference: 998763

Additional supporting information:  crystallographic information; 3D view; checkCIF report

## Figures and Tables

**Table 1:** Hydrogen-bond geometry (Å, °)

*D*—H⋯*A*	*D*—H	H⋯*A*	*D*⋯*A*	*D*—H⋯*A*
C13—H13⋯Br3^i^	0.93	2.85	3.433 (4)	122
C26—H26⋯N4^ii^	0.93	2.62	3.513 (4)	161

Symmetry codes: (i) 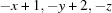; (ii) 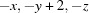.

**Fig. 1.**
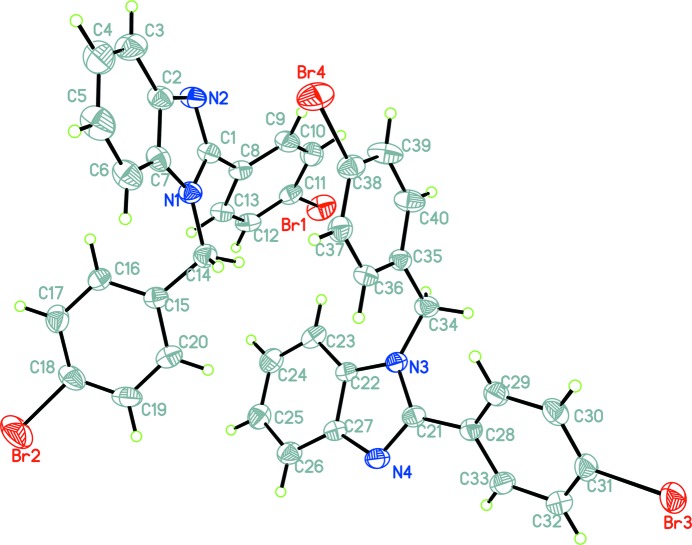
A view of the asymmetric unit of the title compoud with atomic numbering scheme. Displacement ellipsoids were drawn at the 30% probability level. [Symmetry codes: (i) x, y, z; (ii) -x, -y, -z].

## supplementary crystallographic information

### 1. Comment

Organic ligands containing multiple heterocyclic rings are very useful tools in the self-assembly of metallosupramolecular compounds (Steel, 1990). Functionalized benzimidazoles represent an important class of N-containing heterocyclic compounds and have received considerable attention in recent times because their derivatives bear versatile pharmacological properties (Bhattacharya & Chaudhuri, 2008) based on their presence in both clinical medicines (Horton *et al.*, 2003) and compounds of broad biological functions (Boiani *et al.*, 2005). They are important intermediates in many organic reactions (Bai *et al.*, 2001; Hasegawa *et al.*, 1999), and act as ligands to transition metals for modelling biological systems (Bouwman *et al.*, 1990; Pujar *et al.*, 1988). Herein, we report the synthesis and crystal structure of a new benzimidazole derivative 1-(4-bromobenzyl)-2-(4-bromophenyl)-1*H*-benzimidazole (Fig. 1)·In the title compound C_20_H_14_N_2_Br_2_, there are two molecules in the asymmetric unit. The dihedral angles between the least-squares plane of the benzimidazole group and those of the 4-bromobenzyl and 4-chlorophenyl groups are 50.72 (17); 42.09 (16) and 71.29 (16);89.05 (17) respectively. The 4-bromobenzyl and 4-bromophenyl groups make an angle of 68.1 (2); 85.1 (2) (1) with each other. Weak intramolecular hydrogen bonds of C—H···N and Br···Br interactions are observed.

### 2. Experimental

1.0 mmol 4-bromobenzaldehyde and 2 ml water were located in a round bottom flask, and 0.5 mmol benzene-1, 2-diamine was then added. Finally, 0.5 mmol TMSCl was injected to the mixture. The reaction was stirred at room temperature for 5 h to form homogeneous suspension. The suspension was then filtered and the residue was washed with 10 ml water to give product. The crude product was recrystallized with ethanol.

### 3. Refinement

All H atoms were placed in calculated positions with C(sp3)—H = 0.97 and C(sp2)—H = 0.93 \%A, and torsion angles were refined. In the absence of significant anomalous scattering effects, Friedel pairs were averaged.

### Crystal data

**Table:**

C_20_H_14_Br_2_N_2_	*V* = 1756.6 (3) Å^3^
*M**_r_* = 442.15	*Z* = 4
Triclinic, *P*1	*F*(000) = 872
Hall symbol: -P 1	*D*_x_ = 1.672 Mg m^−^^3^
*a* = 9.7537 (9) Å	Mo *K*α radiation, λ = 0.71073 Å
*b* = 10.5758 (10) Å	µ = 4.62 mm^−^^1^
*c* = 17.8255 (17) Å	*T* = 293 K
α = 83.435 (2)°	Block, colorless
β = 81.702 (2)°	0.30 × 0.26 × 0.20 mm
γ = 75.621 (2)°	

### Data collection

**Table:**

Bruker APEXII CCD diffractometer	8094 independent reflections
Radiation source: fine-focus sealed tube	5171 reflections with *I* > 2σ(*I*)
Graphite monochromator	*R*_int_ = 0.034
φ and ω scans	θ_max_ = 27.6°, θ_min_ = 2.0°
Absorption correction: multi-scan (*SADABS*; Bruker, 2004)	*h* = −12→12
*T*_min_ = 0.258, *T*_max_ = 0.398	*k* = −13→13
22195 measured reflections	*l* = −23→23

### Refinement

**Table:**

Refinement on *F*^2^	Primary atom site location: structure-invariant direct methods
Least-squares matrix: full	Secondary atom site location: difference Fourier map
*R*[*F*^2^ > 2σ(*F*^2^)] = 0.041	Hydrogen site location: inferred from neighbouring sites
*wR*(*F*^2^) = 0.128	H-atom parameters constrained
*S* = 1.03	*w* = 1/[σ^2^(*F*_o_^2^) + (0.0682*P*)^2^ + 0.0252*P*] where *P* = (*F*_o_^2^ + 2*F*_c_^2^)/3
8094 reflections	(Δ/σ)_max_ = 0.001
433 parameters	Δρ_max_ = 0.78 e Å^−^^3^
0 restraints	Δρ_min_ = −0.51 e Å^−^^3^

### Special details

**Table:**

Geometry. All e.s.d.'s (except the e.s.d. in the dihedral angle between two l.s. planes) are estimated using the full covariance matrix. The cell e.s.d.'s are taken into account individually in the estimation of e.s.d.'s in distances, angles and torsion angles; correlations between e.s.d.'s in cell parameters are only used when they are defined by crystal symmetry. An approximate (isotropic) treatment of cell e.s.d.'s is used for estimating e.s.d.'s involving l.s. planes.
Refinement. Refinement of *F*^2^ against ALL reflections. The weighted *R*-factor *wR* and goodness of fit *S* are based on *F*^2^, conventional *R*-factors *R* are based on *F*, with *F* set to zero for negative *F*^2^. The threshold expression of *F*^2^ > σ(*F*^2^) is used only for calculating *R*-factors(gt) *etc*. and is not relevant to the choice of reflections for refinement. *R*-factors based on *F*^2^ are statistically about twice as large as those based on *F*, and *R*- factors based on ALL data will be even larger.

### Fractional atomic coordinates and isotropic or equivalent isotropic displacement parameters (Å^2^)

**Table:**

	*x*	*y*	*z*	*U*_iso_*/*U*_eq_	
Br1	−0.15730 (5)	0.89638 (4)	0.54077 (3)	0.08072 (17)	
Br2	−0.16242 (6)	0.32973 (6)	0.10730 (4)	0.1089 (2)	
N1	0.3305 (3)	0.4230 (3)	0.32967 (15)	0.0433 (6)	
N2	0.3833 (3)	0.3609 (3)	0.44875 (15)	0.0500 (7)	
C1	0.3026 (3)	0.4476 (3)	0.40547 (18)	0.0428 (7)	
C2	0.4706 (3)	0.2743 (3)	0.3994 (2)	0.0501 (8)	
C3	0.5784 (4)	0.1635 (4)	0.4142 (2)	0.0675 (11)	
H3	0.5986	0.1356	0.4636	0.081*	
C4	0.6537 (4)	0.0968 (4)	0.3541 (3)	0.0782 (13)	
H4	0.7261	0.0228	0.3629	0.094*	
C5	0.6235 (4)	0.1382 (4)	0.2798 (3)	0.0742 (12)	
H5	0.6784	0.0922	0.2400	0.089*	
C6	0.5146 (4)	0.2452 (4)	0.2633 (2)	0.0621 (10)	
H6	0.4926	0.2709	0.2140	0.075*	
C7	0.4402 (3)	0.3118 (3)	0.32494 (19)	0.0471 (8)	
C8	0.1638 (3)	0.4571 (3)	0.23018 (17)	0.0411 (7)	
C9	0.1261 (3)	0.3402 (3)	0.25350 (19)	0.0478 (8)	
H9	0.1660	0.2877	0.2940	0.057*	
C10	0.0299 (4)	0.3001 (4)	0.2176 (2)	0.0602 (10)	
H10	0.0055	0.2207	0.2331	0.072*	
C11	−0.0294 (4)	0.3804 (4)	0.1582 (2)	0.0625 (10)	
C12	0.0032 (4)	0.4980 (4)	0.1359 (2)	0.0652 (10)	
H12	−0.0391	0.5520	0.0965	0.078*	
C13	0.0992 (4)	0.5355 (4)	0.1723 (2)	0.0547 (9)	
H13	0.1211	0.6161	0.1574	0.066*	
C14	0.1930 (3)	0.5584 (3)	0.43392 (17)	0.0431 (7)	
C15	0.2257 (3)	0.6373 (3)	0.4824 (2)	0.0525 (8)	
H15	0.3189	0.6218	0.4937	0.063*	
C16	0.1234 (4)	0.7384 (4)	0.5144 (2)	0.0584 (9)	
H16	0.1461	0.7903	0.5473	0.070*	
C17	−0.0147 (3)	0.7600 (3)	0.49573 (19)	0.0495 (8)	
C18	−0.0515 (3)	0.6844 (3)	0.44837 (19)	0.0523 (9)	
H18	−0.1451	0.6999	0.4377	0.063*	
C19	0.0526 (3)	0.5847 (3)	0.41662 (18)	0.0479 (8)	
H19	0.0292	0.5341	0.3832	0.057*	
C20	0.2732 (3)	0.5045 (3)	0.26516 (18)	0.0477 (8)	
H20A	0.3518	0.5118	0.2261	0.057*	
H20B	0.2297	0.5917	0.2810	0.057*	
Br3	0.63928 (5)	1.35816 (5)	−0.04992 (2)	0.07448 (16)	
Br4	0.74016 (4)	0.49101 (4)	0.33062 (3)	0.07748 (16)	
N3	0.2139 (3)	0.9848 (2)	0.18779 (14)	0.0400 (6)	
N4	0.1287 (3)	1.0167 (3)	0.07537 (15)	0.0472 (6)	
C21	0.2261 (3)	1.0367 (3)	0.11357 (17)	0.0417 (7)	
C22	0.0980 (3)	0.9287 (3)	0.19718 (17)	0.0403 (7)	
C23	0.0326 (4)	0.8664 (3)	0.2605 (2)	0.0530 (9)	
H23	0.0673	0.8531	0.3075	0.064*	
C24	−0.0858 (4)	0.8261 (4)	0.2497 (2)	0.0593 (9)	
H24	−0.1333	0.7851	0.2907	0.071*	
C25	−0.1374 (4)	0.8444 (4)	0.1792 (2)	0.0605 (10)	
H25	−0.2176	0.8148	0.1744	0.073*	
C26	−0.0721 (3)	0.9056 (4)	0.1164 (2)	0.0542 (9)	
H26	−0.1060	0.9171	0.0694	0.065*	
C27	0.0471 (3)	0.9493 (3)	0.12673 (18)	0.0441 (7)	
C28	0.3296 (3)	1.1121 (3)	0.07887 (17)	0.0428 (7)	
C29	0.4738 (3)	1.0776 (3)	0.08827 (19)	0.0498 (8)	
H29	0.5086	1.0045	0.1204	0.060*	
C30	0.5661 (4)	1.1508 (4)	0.0504 (2)	0.0526 (8)	
H30	0.6623	1.1272	0.0568	0.063*	
C31	0.5138 (4)	1.2583 (4)	0.00333 (19)	0.0518 (8)	
C32	0.3721 (4)	1.2963 (4)	−0.0070 (2)	0.0583 (9)	
H32	0.3386	1.3704	−0.0387	0.070*	
C33	0.2796 (4)	1.2225 (3)	0.03065 (19)	0.0523 (8)	
H33	0.1836	1.2468	0.0237	0.063*	
C34	0.4011 (3)	0.8685 (3)	0.26853 (17)	0.0405 (7)	
C35	0.4468 (4)	0.8454 (4)	0.33915 (19)	0.0593 (10)	
H35	0.4098	0.9070	0.3745	0.071*	
C36	0.5462 (4)	0.7330 (4)	0.3592 (2)	0.0663 (11)	
H36	0.5747	0.7180	0.4077	0.080*	
C37	0.6015 (3)	0.6445 (3)	0.3062 (2)	0.0499 (8)	
C38	0.5589 (3)	0.6633 (3)	0.23460 (19)	0.0490 (8)	
H38	0.5971	0.6018	0.1992	0.059*	
C39	0.4581 (3)	0.7755 (3)	0.21666 (18)	0.0468 (8)	
H39	0.4277	0.7890	0.1686	0.056*	
C40	0.2950 (3)	0.9941 (3)	0.24839 (18)	0.0459 (8)	
H40A	0.2292	1.0191	0.2934	0.055*	
H40B	0.3456	1.0628	0.2331	0.055*	

### Atomic displacement parameters (Å^2^)

**Table:**

	*U*^11^	*U*^22^	*U*^33^	*U*^12^	*U*^13^	*U*^23^
Br1	0.0788 (3)	0.0672 (3)	0.0755 (3)	0.0185 (2)	0.0038 (2)	−0.0161 (2)
Br2	0.0928 (4)	0.1135 (4)	0.1374 (5)	−0.0157 (3)	−0.0652 (3)	−0.0351 (4)
N1	0.0403 (14)	0.0471 (15)	0.0417 (15)	−0.0073 (11)	−0.0073 (11)	−0.0042 (12)
N2	0.0452 (15)	0.0564 (17)	0.0450 (15)	−0.0001 (13)	−0.0116 (12)	−0.0090 (13)
C1	0.0391 (16)	0.0477 (19)	0.0423 (17)	−0.0088 (14)	−0.0080 (13)	−0.0056 (14)
C2	0.0403 (17)	0.052 (2)	0.056 (2)	−0.0013 (15)	−0.0109 (15)	−0.0112 (16)
C3	0.055 (2)	0.065 (2)	0.076 (3)	0.0094 (19)	−0.0206 (19)	−0.012 (2)
C4	0.060 (2)	0.062 (3)	0.108 (4)	0.009 (2)	−0.017 (2)	−0.031 (2)
C5	0.061 (2)	0.079 (3)	0.077 (3)	0.003 (2)	−0.001 (2)	−0.038 (2)
C6	0.057 (2)	0.070 (3)	0.059 (2)	−0.0081 (19)	−0.0032 (17)	−0.0253 (19)
C7	0.0357 (16)	0.054 (2)	0.053 (2)	−0.0091 (14)	−0.0043 (14)	−0.0145 (16)
C8	0.0412 (16)	0.0445 (18)	0.0340 (16)	−0.0038 (13)	−0.0007 (13)	−0.0067 (13)
C9	0.0463 (18)	0.0428 (19)	0.052 (2)	−0.0029 (14)	−0.0135 (15)	−0.0013 (15)
C10	0.052 (2)	0.047 (2)	0.083 (3)	−0.0071 (16)	−0.0150 (19)	−0.0115 (19)
C11	0.048 (2)	0.070 (3)	0.071 (3)	−0.0017 (18)	−0.0218 (18)	−0.022 (2)
C12	0.067 (2)	0.075 (3)	0.051 (2)	−0.007 (2)	−0.0215 (18)	0.0022 (19)
C13	0.056 (2)	0.053 (2)	0.050 (2)	−0.0093 (16)	−0.0040 (16)	0.0083 (16)
C14	0.0463 (17)	0.0454 (18)	0.0344 (16)	−0.0051 (14)	−0.0062 (13)	−0.0010 (13)
C15	0.0433 (18)	0.057 (2)	0.056 (2)	−0.0050 (15)	−0.0127 (15)	−0.0088 (17)
C16	0.063 (2)	0.052 (2)	0.060 (2)	−0.0064 (17)	−0.0101 (18)	−0.0136 (17)
C17	0.0503 (19)	0.0428 (18)	0.0465 (19)	0.0003 (15)	0.0011 (15)	0.0001 (15)
C18	0.0434 (18)	0.058 (2)	0.0468 (19)	0.0004 (15)	−0.0058 (15)	0.0036 (16)
C19	0.0468 (18)	0.053 (2)	0.0421 (18)	−0.0064 (15)	−0.0083 (14)	−0.0032 (15)
C20	0.0546 (19)	0.0498 (19)	0.0389 (17)	−0.0142 (15)	−0.0045 (14)	−0.0022 (14)
Br3	0.0869 (3)	0.1047 (4)	0.0508 (2)	−0.0628 (3)	−0.0016 (2)	−0.0045 (2)
Br4	0.0602 (3)	0.0730 (3)	0.0883 (3)	0.0071 (2)	−0.0228 (2)	0.0056 (2)
N3	0.0405 (13)	0.0419 (14)	0.0366 (14)	−0.0078 (11)	−0.0078 (10)	0.0007 (11)
N4	0.0477 (15)	0.0530 (17)	0.0406 (15)	−0.0120 (12)	−0.0097 (12)	0.0033 (12)
C21	0.0403 (16)	0.0435 (18)	0.0386 (17)	−0.0061 (13)	−0.0054 (13)	0.0004 (13)
C22	0.0399 (16)	0.0381 (17)	0.0387 (17)	−0.0034 (13)	−0.0041 (13)	0.0010 (13)
C23	0.059 (2)	0.0447 (19)	0.0473 (19)	−0.0027 (16)	−0.0013 (16)	0.0022 (15)
C24	0.052 (2)	0.057 (2)	0.063 (2)	−0.0135 (17)	0.0059 (17)	0.0041 (18)
C25	0.0438 (19)	0.058 (2)	0.079 (3)	−0.0147 (17)	−0.0080 (18)	0.005 (2)
C26	0.0481 (19)	0.059 (2)	0.057 (2)	−0.0134 (16)	−0.0150 (16)	0.0036 (17)
C27	0.0417 (17)	0.0449 (18)	0.0428 (18)	−0.0078 (14)	−0.0041 (14)	0.0024 (14)
C28	0.0454 (17)	0.0474 (18)	0.0381 (17)	−0.0148 (14)	−0.0061 (13)	−0.0033 (14)
C29	0.0490 (19)	0.051 (2)	0.0502 (19)	−0.0103 (16)	−0.0106 (15)	−0.0028 (16)
C30	0.0422 (17)	0.068 (2)	0.053 (2)	−0.0191 (16)	−0.0050 (15)	−0.0127 (18)
C31	0.058 (2)	0.065 (2)	0.0413 (18)	−0.0330 (18)	0.0018 (15)	−0.0090 (16)
C32	0.068 (2)	0.058 (2)	0.051 (2)	−0.0204 (19)	−0.0123 (18)	0.0050 (17)
C33	0.0518 (19)	0.057 (2)	0.049 (2)	−0.0173 (16)	−0.0104 (15)	0.0078 (16)
C34	0.0406 (16)	0.0464 (18)	0.0355 (16)	−0.0124 (13)	−0.0041 (12)	−0.0032 (13)
C35	0.058 (2)	0.078 (3)	0.0351 (18)	0.0038 (18)	−0.0091 (15)	−0.0170 (17)
C36	0.057 (2)	0.094 (3)	0.0408 (19)	0.001 (2)	−0.0134 (16)	−0.0055 (19)
C37	0.0405 (17)	0.054 (2)	0.052 (2)	−0.0066 (15)	−0.0065 (15)	0.0020 (16)
C38	0.0515 (19)	0.0449 (19)	0.0494 (19)	−0.0077 (15)	−0.0072 (15)	−0.0060 (15)
C39	0.0542 (19)	0.0493 (19)	0.0395 (17)	−0.0134 (15)	−0.0133 (14)	−0.0022 (14)
C40	0.0537 (19)	0.0464 (19)	0.0378 (17)	−0.0095 (15)	−0.0083 (14)	−0.0053 (14)

### Geometric parameters (Å, º)

**Table:**

Br1—C17	1.899 (3)	Br3—C31	1.895 (3)
Br2—C11	1.895 (4)	Br4—C37	1.890 (3)
N1—C1	1.381 (4)	N3—C21	1.374 (4)
N1—C7	1.381 (4)	N3—C22	1.383 (4)
N1—C20	1.448 (4)	N3—C40	1.453 (4)
N2—C1	1.308 (4)	N4—C21	1.316 (4)
N2—C2	1.385 (4)	N4—C27	1.379 (4)
C1—C14	1.460 (4)	C21—C28	1.464 (5)
C2—C7	1.394 (5)	C22—C27	1.391 (4)
C2—C3	1.395 (4)	C22—C23	1.395 (4)
C3—C4	1.368 (6)	C23—C24	1.372 (5)
C3—H3	0.9300	C23—H23	0.9300
C4—C5	1.397 (6)	C24—C25	1.396 (5)
C4—H4	0.9300	C24—H24	0.9300
C5—C6	1.382 (5)	C25—C26	1.382 (5)
C5—H5	0.9300	C25—H25	0.9300
C6—C7	1.386 (5)	C26—C27	1.395 (5)
C6—H6	0.9300	C26—H26	0.9300
C8—C13	1.372 (4)	C28—C29	1.392 (4)
C8—C9	1.378 (5)	C28—C33	1.396 (4)
C8—C20	1.519 (5)	C29—C30	1.384 (5)
C9—C10	1.379 (5)	C29—H29	0.9300
C9—H9	0.9300	C30—C31	1.369 (5)
C10—C11	1.380 (5)	C30—H30	0.9300
C10—H10	0.9300	C31—C32	1.373 (5)
C11—C12	1.361 (6)	C32—C33	1.389 (5)
C12—C13	1.368 (5)	C32—H32	0.9300
C12—H12	0.9300	C33—H33	0.9300
C13—H13	0.9300	C34—C35	1.371 (4)
C14—C15	1.387 (5)	C34—C39	1.386 (4)
C14—C19	1.400 (4)	C34—C40	1.509 (4)
C15—C16	1.382 (5)	C35—C36	1.382 (5)
C15—H15	0.9300	C35—H35	0.9300
C16—C17	1.392 (5)	C36—C37	1.366 (5)
C16—H16	0.9300	C36—H36	0.9300
C17—C18	1.366 (5)	C37—C38	1.376 (5)
C18—C19	1.380 (5)	C38—C39	1.379 (4)
C18—H18	0.9300	C38—H38	0.9300
C19—H19	0.9300	C39—H39	0.9300
C20—H20A	0.9700	C40—H40A	0.9700
C20—H20B	0.9700	C40—H40B	0.9700
			
C1—N1—C7	106.3 (2)	C21—N3—C22	106.3 (2)
C1—N1—C20	128.2 (3)	C21—N3—C40	128.8 (3)
C7—N1—C20	125.0 (3)	C22—N3—C40	124.7 (2)
C1—N2—C2	104.9 (3)	C21—N4—C27	105.3 (3)
N2—C1—N1	113.1 (3)	N4—C21—N3	112.6 (3)
N2—C1—C14	123.8 (3)	N4—C21—C28	121.6 (3)
N1—C1—C14	123.1 (3)	N3—C21—C28	125.7 (3)
N2—C2—C7	110.5 (3)	N3—C22—C27	105.6 (3)
N2—C2—C3	130.0 (3)	N3—C22—C23	132.0 (3)
C7—C2—C3	119.5 (3)	C27—C22—C23	122.4 (3)
C4—C3—C2	118.4 (4)	C24—C23—C22	116.2 (3)
C4—C3—H3	120.8	C24—C23—H23	121.9
C2—C3—H3	120.8	C22—C23—H23	121.9
C3—C4—C5	121.0 (4)	C23—C24—C25	122.2 (3)
C3—C4—H4	119.5	C23—C24—H24	118.9
C5—C4—H4	119.5	C25—C24—H24	118.9
C6—C5—C4	122.1 (4)	C26—C25—C24	121.5 (3)
C6—C5—H5	118.9	C26—C25—H25	119.2
C4—C5—H5	118.9	C24—C25—H25	119.2
C5—C6—C7	115.9 (4)	C25—C26—C27	117.1 (3)
C5—C6—H6	122.0	C25—C26—H26	121.5
C7—C6—H6	122.0	C27—C26—H26	121.5
N1—C7—C6	131.7 (3)	N4—C27—C22	110.2 (3)
N1—C7—C2	105.2 (3)	N4—C27—C26	129.3 (3)
C6—C7—C2	123.0 (3)	C22—C27—C26	120.6 (3)
C13—C8—C9	118.5 (3)	C29—C28—C33	118.7 (3)
C13—C8—C20	117.9 (3)	C29—C28—C21	124.4 (3)
C9—C8—C20	123.7 (3)	C33—C28—C21	116.9 (3)
C8—C9—C10	121.0 (3)	C30—C29—C28	120.8 (3)
C8—C9—H9	119.5	C30—C29—H29	119.6
C10—C9—H9	119.5	C28—C29—H29	119.6
C9—C10—C11	118.5 (4)	C31—C30—C29	119.1 (3)
C9—C10—H10	120.7	C31—C30—H30	120.4
C11—C10—H10	120.7	C29—C30—H30	120.4
C12—C11—C10	121.3 (3)	C30—C31—C32	121.9 (3)
C12—C11—Br2	118.6 (3)	C30—C31—Br3	119.6 (3)
C10—C11—Br2	120.1 (3)	C32—C31—Br3	118.5 (3)
C11—C12—C13	119.0 (3)	C31—C32—C33	119.0 (3)
C11—C12—H12	120.5	C31—C32—H32	120.5
C13—C12—H12	120.5	C33—C32—H32	120.5
C12—C13—C8	121.6 (4)	C32—C33—C28	120.5 (3)
C12—C13—H13	119.2	C32—C33—H33	119.7
C8—C13—H13	119.2	C28—C33—H33	119.7
C15—C14—C19	118.3 (3)	C35—C34—C39	118.0 (3)
C15—C14—C1	119.6 (3)	C35—C34—C40	120.1 (3)
C19—C14—C1	122.0 (3)	C39—C34—C40	121.9 (3)
C16—C15—C14	121.7 (3)	C34—C35—C36	121.9 (3)
C16—C15—H15	119.2	C34—C35—H35	119.1
C14—C15—H15	119.2	C36—C35—H35	119.1
C15—C16—C17	117.8 (3)	C37—C36—C35	118.4 (3)
C15—C16—H16	121.1	C37—C36—H36	120.8
C17—C16—H16	121.1	C35—C36—H36	120.8
C18—C17—C16	122.4 (3)	C36—C37—C38	121.8 (3)
C18—C17—Br1	119.3 (3)	C36—C37—Br4	119.8 (3)
C16—C17—Br1	118.3 (3)	C38—C37—Br4	118.4 (3)
C17—C18—C19	118.8 (3)	C37—C38—C39	118.4 (3)
C17—C18—H18	120.6	C37—C38—H38	120.8
C19—C18—H18	120.6	C39—C38—H38	120.8
C18—C19—C14	121.0 (3)	C38—C39—C34	121.5 (3)
C18—C19—H19	119.5	C38—C39—H39	119.2
C14—C19—H19	119.5	C34—C39—H39	119.2
N1—C20—C8	115.7 (3)	N3—C40—C34	113.7 (3)
N1—C20—H20A	108.4	N3—C40—H40A	108.8
C8—C20—H20A	108.4	C34—C40—H40A	108.8
N1—C20—H20B	108.4	N3—C40—H40B	108.8
C8—C20—H20B	108.4	C34—C40—H40B	108.8
H20A—C20—H20B	107.4	H40A—C40—H40B	107.7
			
C2—N2—C1—N1	0.5 (4)	C27—N4—C21—N3	0.5 (3)
C2—N2—C1—C14	179.8 (3)	C27—N4—C21—C28	−176.9 (3)
C7—N1—C1—N2	−1.0 (4)	C22—N3—C21—N4	−0.7 (3)
C20—N1—C1—N2	−173.0 (3)	C40—N3—C21—N4	−175.6 (3)
C7—N1—C1—C14	179.7 (3)	C22—N3—C21—C28	176.6 (3)
C20—N1—C1—C14	7.7 (5)	C40—N3—C21—C28	1.6 (5)
C1—N2—C2—C7	0.1 (4)	C21—N3—C22—C27	0.6 (3)
C1—N2—C2—C3	179.3 (4)	C40—N3—C22—C27	175.8 (3)
N2—C2—C3—C4	−177.4 (4)	C21—N3—C22—C23	−177.3 (3)
C7—C2—C3—C4	1.7 (6)	C40—N3—C22—C23	−2.1 (5)
C2—C3—C4—C5	−0.2 (7)	N3—C22—C23—C24	177.6 (3)
C3—C4—C5—C6	−1.8 (7)	C27—C22—C23—C24	0.1 (5)
C4—C5—C6—C7	2.1 (6)	C22—C23—C24—C25	0.8 (5)
C1—N1—C7—C6	−177.4 (4)	C23—C24—C25—C26	−0.5 (6)
C20—N1—C7—C6	−5.1 (6)	C24—C25—C26—C27	−0.6 (5)
C1—N1—C7—C2	1.0 (4)	C21—N4—C27—C22	−0.1 (4)
C20—N1—C7—C2	173.3 (3)	C21—N4—C27—C26	178.8 (3)
C5—C6—C7—N1	177.6 (4)	N3—C22—C27—N4	−0.3 (3)
C5—C6—C7—C2	−0.5 (6)	C23—C22—C27—N4	177.8 (3)
N2—C2—C7—N1	−0.7 (4)	N3—C22—C27—C26	−179.3 (3)
C3—C2—C7—N1	−180.0 (3)	C23—C22—C27—C26	−1.1 (5)
N2—C2—C7—C6	177.9 (3)	C25—C26—C27—N4	−177.4 (3)
C3—C2—C7—C6	−1.4 (6)	C25—C26—C27—C22	1.4 (5)
C13—C8—C9—C10	2.6 (5)	N4—C21—C28—C29	−137.9 (3)
C20—C8—C9—C10	−177.6 (3)	N3—C21—C28—C29	45.0 (5)
C8—C9—C10—C11	−0.8 (5)	N4—C21—C28—C33	39.1 (4)
C9—C10—C11—C12	−1.3 (6)	N3—C21—C28—C33	−137.9 (3)
C9—C10—C11—Br2	−179.9 (3)	C33—C28—C29—C30	−0.3 (5)
C10—C11—C12—C13	1.4 (6)	C21—C28—C29—C30	176.7 (3)
Br2—C11—C12—C13	−180.0 (3)	C28—C29—C30—C31	0.1 (5)
C11—C12—C13—C8	0.5 (6)	C29—C30—C31—C32	0.4 (5)
C9—C8—C13—C12	−2.5 (5)	C29—C30—C31—Br3	−179.2 (3)
C20—C8—C13—C12	177.7 (3)	C30—C31—C32—C33	−0.8 (6)
N2—C1—C14—C15	49.0 (5)	Br3—C31—C32—C33	178.8 (3)
N1—C1—C14—C15	−131.8 (3)	C31—C32—C33—C28	0.6 (5)
N2—C1—C14—C19	−128.0 (4)	C29—C28—C33—C32	−0.1 (5)
N1—C1—C14—C19	51.2 (5)	C21—C28—C33—C32	−177.3 (3)
C19—C14—C15—C16	1.1 (5)	C39—C34—C35—C36	−0.3 (6)
C1—C14—C15—C16	−176.1 (3)	C40—C34—C35—C36	−178.5 (4)
C14—C15—C16—C17	−0.7 (5)	C34—C35—C36—C37	1.2 (6)
C15—C16—C17—C18	0.9 (5)	C35—C36—C37—C38	−1.3 (6)
C15—C16—C17—Br1	178.8 (3)	C35—C36—C37—Br4	178.9 (3)
C16—C17—C18—C19	−1.4 (5)	C36—C37—C38—C39	0.4 (5)
Br1—C17—C18—C19	−179.3 (2)	Br4—C37—C38—C39	−179.7 (3)
C17—C18—C19—C14	1.7 (5)	C37—C38—C39—C34	0.6 (5)
C15—C14—C19—C18	−1.6 (5)	C35—C34—C39—C38	−0.6 (5)
C1—C14—C19—C18	175.5 (3)	C40—C34—C39—C38	177.6 (3)
C1—N1—C20—C8	−107.0 (4)	C21—N3—C40—C34	−106.7 (3)
C7—N1—C20—C8	82.3 (4)	C22—N3—C40—C34	79.2 (4)
C13—C8—C20—N1	175.6 (3)	C35—C34—C40—N3	−156.4 (3)
C9—C8—C20—N1	−4.2 (4)	C39—C34—C40—N3	25.4 (4)

### Hydrogen-bond geometry (Å, º)

**Table:**

*D*—H···*A*	*D*—H	H···*A*	*D*···*A*	*D*—H···*A*
C13—H13···Br3^i^	0.93	2.85	3.433 (4)	122
C26—H26···N4^ii^	0.93	2.62	3.513 (4)	161

Symmetry codes: (i) −*x*+1, −*y*+2, −*z*; (ii) −*x*, −*y*+2, −*z*.
